# Regional Disparities and Associated Factors Underlying CDC Health Professional Distribution in China

**DOI:** 10.3390/healthcare14081079

**Published:** 2026-04-17

**Authors:** Jiayi Zheng, Tong Hu, Shandan Xu, Jing Xiao, Change Xiong

**Affiliations:** 1 School of Public Health, Wuhan University of Science and Technology, Wuhan 430065, China; jiayi0509@wust.edu.cn (J.Z.); ht0601@wust.edu.cn (T.H.); xushandan@wust.edu.cn (S.X.); 2 Hubei Provincial Center for Disease Control and Prevention, Wuhan 430079, China

**Keywords:** health professionals, disparity, spatial agglomeration, Moran’s index, geographic detector

## Abstract

**Aim:** The aim of this study was to explore the distribution and driving factors influencing the disparity of health professionals (HPs) at the Centers for Disease Control and Prevention (CDC) in China and to provide a reference for regional health planning and rational allocation of public health resources. **Methods:** The Gini coefficient was used to measure the equity of HP distribution at CDC sites at the provincial level during 2012–2023 in China. Moran’s I was used to analyze the spatial agglomeration effect, and the geographic detector model was used to explore the factors driving the allocation of HPs at CDC sites in different provinces. **Results:** The number of HPs at the CDC showed an increasing trend from 2012 to 2023 in China. The average Gini coefficients at the population and geographical areas were 0.16 and 0.58, respectively. The global Moran’s I statistic indicated a notable decline in spatial clustering for the population dimension, decreasing from 0.503 to 0.238; in contrast, spatial clustering for the geographical dimension remained relatively stable, ranging between 0.13 and 0.16. The local Moran’s I statistic revealed that provinces such as Qinghai in the western China consistently exhibited a “low–low” spatial clustering pattern. Six factors were found to explain the variability in the CDC HP distribution based on the 2020 data. In the context of factor interactions, the synergistic effects between education level and health expenditure share (q = 0.9781), and between population aging and per capita GDP (q = 0.9699), substantially exceed the explanatory power attributable to any single factor alone. **Conclusions:** A significant regional disparity was observed in the distribution of HPs among 31 provinces, with the distribution based on service area being less equitable than that based on population. The shortage of healthcare professionals in the western region is characterized by notably inadequate geographical distribution. Future policy initiatives should prioritize targeted spatial interventions and integrated, multi-factor collaborative strategies.

## 1. Introduction

Equitable allocation of health resources is one of the basic conditions to ensure the equity of health services at the population level [1]. It is also one of the main objectives of government interventions in the health services market. With the rapid growth of the economy, China’s health spending has increased dramatically [2]. However, inadequate and unbalanced public health resources are still a challenge. Health professionals (HPs) at the Centers for Disease Control and Prevention (CDC) in China include practitioners, registered nurses, pharmacists, laboratory technicians, imaging technicians and other categories, excluding those engaged in administrative tasks [3]. An evaluation of the equity of CDC health personnel distribution is in line with the strategic goals of Healthy China 2030 and the ongoing reforms involving disease prevention and control strategies.

Existing studies have mainly focused on the distribution of medical resources, rather than public health. However, the medical system and the disease prevention and control system are equally important in China. The CDC is the backbone of the disease prevention and control system in China. It has a vital responsibility in promoting people’s health, ensuring public health security, and maintaining economic and social stability. However, there are differences between China’s CDC and those in other countries in terms of institutional nature, organizational structure and work functions. For example, the CDC in the United States is a government agency while the CDC in China is a public institution, and the U.S. CDC works more broadly than the Chinese CDC. There are four levels of CDC in China; namely, the national, provincial, city and county levels. Since the COVID-19 outbreak, the CDC has required a growing number of skilled health professionals given its increasingly important role in disease prevention in China. However, there are few studies on the distribution of CDC personnel, and these are limited to the provincial and city levels. There is a lack of nationwide analysis of CDC personnel distribution. At the same time, relevant factors affecting the distribution of CDC health professionals are also rarely reported, and there is a lack of evidence for optimization of the distribution of health professionals in the CDC. Elucidating the distribution characteristics and influencing factors of CDC health professionals is the basis of high-quality development of the disease control system.

Several studies have described methods to evaluate inequity. The study reported by McGrail measured primary healthcare (PHC) accessibility in rural Australia using a new index approach. The primary methodological advantage of this approach resides in its explicit integration of spatial alignment between service delivery and population demand [4]. Kharazmi investigated the distribution of the nursing workforce around the world using the Gini coefficient and Lorenz curve, thereby empirically confirming substantial inequities in the geographical allocation of nursing personnel worldwide [5]. The regional disparity and the factors influencing the distribution of high-quality medical resources (HQMRs) were investigated in another study, which represents the first systematic investigation into how multivariate interactions influence the allocation of health resources [6]. Wu et al. conducted a longitudinal analysis of population-weighted and geographically weighted distribution patterns of health professionals across China from 2002 to 2016, employing widely accepted inequality metrics—including the Gini coefficient and the Theil index—to quantify disparities. Their findings indicate that, while the health workforce distribution exhibits relative equity when adjusted for population size, it demonstrates pronounced spatial inequity across administrative regions. This nuanced characterization of China’s health resource allocation has since served as a foundational reference for policy-oriented and equity-focused health systems research [7]. However, these methodologies have been predominantly employed in the assessment of healthcare accessibility, whereas their application to the analysis of health human resources—particularly within disease prevention and control—remains limited.

In recent years, scholars worldwide have employed diverse methodological approaches to investigate strategies for optimizing the allocation of health workforce resources. Domestic research has primarily focused on proposing policy recommendations grounded in considerations of equity and fairness. Zhang et al. identified two interrelated challenges undermining the effectiveness of epidemic response: the uneven geographical distribution of public health personnel and the consequent widening of regional disparities in outbreak control capacity. To address these issues, the authors proposed a three-pronged intervention strategy, comprising (1) performance-linked salary incentives to enhance workforce retention in underserved areas; (2) an AI-driven dynamic personnel allocation model to optimize real-time deployment based on epidemiological risk and resource availability; and (3) a standardized cross-regional competency development program to strengthen institutional capacity across jurisdictions [8]. Upon identifying a critical shortage of human resources in western China, Zhou proposed that staffing allocation criteria should comprehensively integrate demographic and geographic considerations, and that targeted policy adjustments be implemented to improve the competitiveness and appeal of positions in the region [9]. Shao et al. proposed the development of tiered resource allocation standards calibrated to regional economic development levels, advocating for increased investment in underdeveloped areas and the implementation of enhanced retention mechanisms for key personnel [10]. International scholarly research provides a range of evidence-based perspectives on potential solutions. Naden et al. demonstrated, through a longitudinal early-career intervention program implemented in rural Australia, that targeted academic and vocational support for students during middle school significantly enhances the likelihood of their subsequent recruitment and long-term retention as local healthcare professionals [11]. An Australian policy review underscored the need to establish a comprehensive, end-to-end rural training pathway spanning the entirety of medical education. This includes expanding investment in rural clinical schools, fostering students’ long-term professional identification with rural practice, and reinforcing medical colleges’ social mandate to recruit from—and remain committed to—rural communities [12]. Garg et al. conducted a rigorous empirical analysis of the Indian labor market, systematically identifying key structural determinants—including deficiencies in recruitment practices and insufficient salary competitiveness—as root causes of labor market inefficiencies. Building on these findings, they designed and implemented targeted interventions [13].

The aforementioned studies offer valuable methodological frameworks and empirical insights for the present research; however, several critical gaps remain to be addressed. First, spatial heterogeneity in health resource allocation has not been systematically examined—existing assessments predominantly rely on aggregate fairness metrics, thereby overlooking localized disparities and failing to identify specific geographical areas where inequities arise. Second, the attribution analysis remains underdeveloped: few studies have quantitatively disentangled the relative contributions of multiple determinants—including institutional, socioeconomic and geographic factors—as well as their synergistic effects on the distribution of public health control resources. Third, policy recommendations lack granularity and contextual grounding, with current proposals emphasizing broad, macro-level investment strategies without integrating spatially explicit diagnostics or evidence-based, factor-specific intervention pathways.

This study defines two foundational dimensions of equity in the context of public health workforce distribution: (1) population-based equity, which assesses the proportional alignment between the number of health professionals and the size of the permanent resident population, thereby reflecting service accessibility across demographic groups; and (2) geographical equity, which evaluates the spatial uniformity of HPs density per unit land area, thereby capturing the physical reachability of services. In China, the delineation of public health service grids is a widely adopted administrative strategy to enhance accountability and effectiveness in disease prevention and control. Consequently, analyzing the geographical distribution density of health professionals holds significant policy and operational relevance. Accordingly, this study adopts a dual-dimensional equity assessment framework encompassing both population and geographical perspectives.

In this study, we proposed a three-stage analytical approach. The Gini coefficient was used to evaluate the equity of CDC HP distribution during 2012–2023, followed by spatial correlation analysis to identify specific regions of inequity. The geographical detector method was also used to analyze the factors influencing the allocation of HPs at CDC sites in different provinces. This approach does not entail the development of novel analytical methodologies; rather, it adapts and applies established analytical methods to the domain of public health workforce management. This study not only provides a reference for improving the policies related to CDC HPs, but also offers theoretical support for the development of HPs in China.

## 2. Materials and Methods

### 2.1. Data Source

In this study, the number of HPs at CDC sites was extracted from the 2013–2024 China Health Statistical Yearbook (http://www.nhc.gov.cn/mohwsbwstjxxzx/tjzxtjsj/tjsj_list.shtml, accessed on 31 October 2025). The data pertaining to permanent resident population and geographical area were obtained from the China Statistical Yearbook for the years 2013 to 2024 (https://www.stats.gov.cn/sj/ndsj/, accessed on 31 October 2025). The geographical information was obtained from the website of the National Geomatics Center of China (https://www.ngcc.cn/, accessed on 31 October 2025).

All variable definitions were maintained consistently throughout the study period (2012–2023). Specifically, the indicator “the Number of HPs in CDCs,” as reported in the China Health Statistics Yearbook, encompasses practicing physicians, registered nurses, pharmacists, laboratory technicians, radiological and imaging technicians, and other qualified health professionals; personnel engaged exclusively in administrative or managerial functions are explicitly excluded. This standardized definition was applied uniformly across all provinces and annual reporting cycles, thereby ensuring temporal and geographical comparability of the data. Furthermore, the dataset exhibited no missing values for this indicator at any time point or jurisdiction.

Based on a review of the relevant literature [14,15,16] and available data, demographic structure, population health status, economic development, and health expenses were selected as influencing factors for analysis. The dependent variables include the total number of HPs, the number of HPs per 1000 persons (distribution based on population) and the number of HPs per square kilometer (distribution based on geographical area).

For detailed information, please refer to Table 1.

### 2.2. Setting

China has a land area of approximately 9.6 million square kilometers, and its population was about 1.4 billion at the end of 2023. A total of 3376 CDC sites existed in China in 2023. A total of 31 provinces, autonomous regions, and municipalities were considered in this study. The publicly available health workforce data are reported exclusively at the provincial level; granular data disaggregated by prefecture-level and county-level administrative units are not currently accessible. This limitation constrains the feasibility of conducting spatially refined analyses. While provincial-level aggregation may obscure intra-provincial heterogeneity in the distribution and composition of health technical personnel, it nonetheless provides an essential baseline for informing future research with higher geographic resolution.

### 2.3. Evaluation Methods

A tripartite methodological framework comprising the Gini coefficient, Moran’s I statistic and a geographical detector was adopted in this study to systematically examine the spatial distribution patterns and underlying mechanisms of health human resource allocation across CDC sites. Specifically, these methods jointly address three complementary analytical dimensions: equity assessment, spatial autocorrelation detection and explanatory factor identification.

The Gini coefficient—a widely validated measure of distributional equity—quantifies cumulative inequality in health personnel allocation relative to both population size and geographical area. Its integration with the Lorenz curve offers enhanced visual interpretability compared to alternative indices (e.g., the Theil index or concentration index), facilitating intuitive assessment of disparities in service accessibility and territorial coverage. By computing separate Gini values for population- and area-based denominators, the analysis enables dual-dimensional evaluation of equity performance.

Moran’s I statistic complements the Gini coefficient by explicitly accounting for spatial structure. The global Moran’s I tests for statistically significant spatial autocorrelation in provincial-level health personnel density, distinguishing between random, clustered and dispersed allocation patterns. The local Moran’s I further pinpoints spatial clusters, thereby identifying priority zones for targeted, region-specific policy interventions.

A geographical detector overcomes key limitations of conventional regression approaches, including restrictive linearity assumptions and sensitivity to multicollinearity, by quantifying both the individual explanatory power (q-statistic) of each determinant and the interactive effects between pairs of factors. This capability enables robust detection of nonlinear relationships, synergistic amplifications and potential antagonisms among socioeconomic, demographic and policy-related drivers.

### 2.4. Gini Coefficient (G)

The Gini coefficient is a quantitative index proposed by Italian economist Corrado Gini to measure the difference in the income distribution of residents in a country (or region) and is frequently combined with the Lorentz curve. The Gini coefficient is calculated according to the size of the area enclosed by the Lorentz curve and the absolute fairness line. The study of equitable allocation of HPs is based on two dimensions of distribution, which represent equality based on population (1000 persons) and geographical (square kilometers) accessibility. The Gini coefficient ranges from 0 to 1 [17]. A smaller value indicates higher equitable resource distribution. A value below 0.2 indicates an absolute balance in resource distribution, while a value over 0.6 indicates a highly unfair resource distribution [18].

### 2.5. Global Moran’s Index and Local Moran’s Index

Global spatial autocorrelation analysis is used to measure the degree of spatial correlation and difference between regions [19]. In this study, the global spatial autocorrelation is based on the global Moran’s I, the value range of which is [−1, 1] [20]. A global Moran’s I value greater than 0 suggests positive spatial correlation of the data, with a value closer to 1 indicating stronger spatial aggregation of resources. A global Moran’s I less than 0 implies spatial negative correlation and, the closer it is to −1, the stronger the spatial dispersion of the resource distribution. A global Moran’s I equal to 0 indicates a lack of spatial autocorrelation [21].

The local Moran’s I was used to analyze the degree of correlation between resource allocation locally in a region and its neighbors. A Local Indicators of Spatial Association (LISA) cluster map of spatial association was used to visualize the agglomeration effect. The analysis of local Moran’s I revealed four significant differences and one insignificant difference. The four significant differences were “high–high”, “high–low”, “low–high” and “low–low” distributions. The “high–high” and “low–low” types represent high/low levels of aggregation of a specific resource in a specific region and the high/low level of resource aggregation in the surrounding region. The “high–low” and “low–high” types represent high/low levels of resource aggregation in a certain region but low/high levels of resource aggregation in the surrounding areas. A statistically non-significant type indicates that the level of resources in a certain region lacks significant correlation with the degree of resource aggregation in the surrounding region, indicating stochastic distribution [22].

The computation of both the global and local Moran’s I statistics relies on a first-order spatial weight matrix constructed under the Queen contiguity criterion. Under this rule, wij = 1 if provinces i and j share either a common boundary or a vertex; otherwise, wij = 0. To address the topological isolation of Hainan Province, an island jurisdiction, its adjacency was explicitly assigned to Guangdong Province based on geographical proximity, consistent with established practices in spatial econometrics for handling non-contiguous units. Subsequently, the weight matrix underwent row-standardization to ensure that the sum of weights for each province’s spatial neighbors equals unity, thereby mitigating potential bias arising from heterogeneous neighborhood sizes or boundary-length variation. Statistical significance was assessed via 999 Monte Carlo random permutations, yielding empirical *p*-values for hypothesis testing.

### 2.6. Geographic Detector

A geographic detector (geodetector) is a tool that utilizes the theory of spatial differentiation to assess the relationship between independent and dependent variables. It analyzes various types of variables on the same spatial scale and can identify the spatial heterogeneity of individual variables [23]. Moreover, interaction detection enables the evaluation of whether the joint explanatory power of any two factors, regarding the spatial heterogeneity of the dependent variable, exceeds the sum of their individual contributions, thereby revealing potential synergistic or antagonistic interactions among the factors. Other commonly used methods, such as logistic regression models, have more restrictions on data distribution and data size than geographic detectors. The geographic detector model is not limited by assumptions of linearity, which means that collinearity among independent variables does not impact the interpretation of the final results. In this study, the relationship between various factors and the distribution of HPs was determined based on factor and interactive detection. Factor detection can be used to detect the extent to which factor X contributes to the spatial differentiation of HP allocation in the CDC. Interactive detection can reveal the relationship between different factors; in particular, to determine whether the combination of factors x1 and x2 increases or decreases the explanatory power of dependent variable Y or whether the effects of these factors on Y are independent of each other. Geographic detectors can be used to measure the explanatory power of independent variables relative to dependent variables with the q value, which ranges from 0 to 1. The larger the value, the stronger the explanatory power of X relative to Y. In this study, the continuous variables x1 through x9 were discretized into four ordinal categories using the K-means clustering algorithm. This unsupervised partitioning approach leverages iterative centroid optimization to group observations with similar values, thereby preserving the underlying distributional characteristics of each variable. All q-statistics reported in the primary analysis were computed based on this consistent discretization framework.

### 2.7. Data Analysis

SPSS 26.0 was used to analyze the HP distribution in mainland China. The Gini coefficient was calculated using Stata16.0. The spatial autocorrelation analysis was conducted and validated using ArcGis 10.8. Geographical detector analysis was performed using the GeoDetector software package, developed by the research team led by Professor Wang Jinfeng at the Institute of Geographic Sciences and Natural Resources Research, Chinese Academy of Sciences (http://www.geodetector.cn/, accessed on 3 December 2025). The tool is implemented as an Excel add-in. *p* < 0.05 is generally interpreted as indicating statistical significance.

## 3. Results

### 3.1. Descriptive Statistics

#### 3.1.1. Temporal Distribution of CDC HPs in China from 2012 to 2023

The results showed an increase in the number and density of CDC HPs in China from 2012 to 2023 (Table 2). The densities of HPs per 1000 persons and per square kilometer increased by 23.82% and 28.77% over the past decade, respectively (Figure S1). It is important to note that the statistical yearbook data employed in this study report only the aggregate number of personnel and do not disaggregate the sources of growth. Given the inherent limitations of publicly available yearbook data in distinguishing these distinct institutional mechanisms, this study presents only the net change in personnel numbers and refrains from attributing growth to specific causes.

#### 3.1.2. Spatial Distribution of CDC HPs in 2023

In order to obtain a deeper insight into the distribution of CDC HPs in China, we calculated the HP density per capita and per square kilometer in every province in 2023. The specific values are presented in Table S1. The number of CDC HPs per 1000 persons in Tibet (0.4132) was about 5.8-fold higher than in Guangdong (0.0713). However, the comparative analysis of the geographical distribution of HPs showed a completely different result. Among all 31 provincial regions, Shanghai ranked first in HPs per square kilometer, followed by Beijing. The difference between the highest (Shanghai, 0.4759) and lowest (Tibet, 0.0012) proportions of health personnel per square kilometer is approximately 396-fold. Geographical density refers solely to the number of healthcare personnel per square kilometer and does not reflect the actual accessibility of health services for residents; therefore, these two concepts are not interchangeable.

A descriptive statistical analysis of driving factors is provided in Table S2.

### 3.2. Disparity of HP Distribution

#### Gini Coefficient (G)

Table 3 shows that the calculated Gini coefficient of HPs allocated by population in 2023 was 0.1624, while that based on geographical region was 0.5848. The Gini coefficient for CDC sites based on population remained stable between 0.15 and 0.17 from 2012 to 2023, which suggests equal distribution of HPs. The Gini coefficient based on geographical area remained stable from 0.5809 in 2012 to 0.5848 in 2023, persistently indicating a high level of spatial inequality (Figure S2). Overall, the Gini coefficient exhibited minimal interannual variation and showed no statistically discernible upward or downward trend.

### 3.3. Spatial Aggregation Analysis of CDC HP Distribution

#### 3.3.1. Global Moran’s I

The global Moran’s I values of HPs in Chinese CDC sites based on population level and geographical area were 0.238 (*p* < 0.05) and 0.151 (*p* < 0.05) in 2023, respectively, indicating significant spatial aggregation. Thus, the regions with abundant (or scarce) CDC health human resources were more clustered in China. As shown in Table 4, the global Moran’s I of HPs by population declined from 0.503 to 0.238 from 2012 to 2023, demonstrating a gradual weakening of the spatial clustering of HPs. The global Moran’s I for geographical distribution showed an overall weak and fluctuating upward trend from 2012 to 2023. The Global Moran’s I of HPs increased from 0.137 to 0.151, implying a more pronounced clustering pattern (Figure S3).

#### 3.3.2. Local Moran’s I

**Figure 1:**
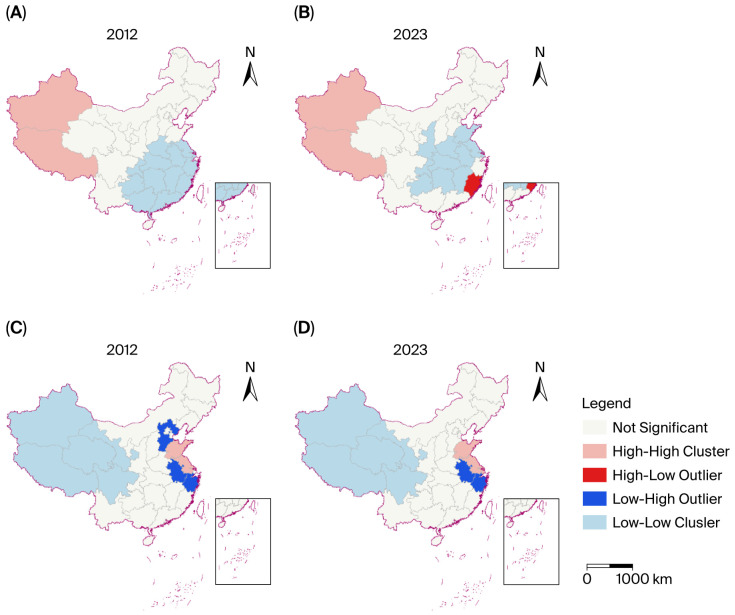
The local Moran’s I of distribution for CDC health professionals in 2012 and 2023 ((**A**,**B**) are based on the population dimension; (**C**,**D**) are based on geographical area dimension). Note: The “high–high” and “low–low” types represent high/low levels of aggregation of a specific resource in a specific region and the high/low level of resource aggregation in the surrounding region. The “high–low” and “low–high” types represent high/low levels of resource aggregation in a certain region but low/high levels of resource aggregation in the surrounding areas. A statistically non-significant type indicates that the level of resources in a certain region lacks significant correlation with the degree of resource aggregation in the surrounding region, indicating stochastic distribution.

Figure 1A and Figure 1B present the distribution of HPs by population in 2012 and 2023, respectively. The high–high type was more prominent in the northwest and southwest regions of China (Xinjiang, Tibet) in 2012. Meanwhile, the provinces that showed low–low type spatial autocorrelation were mainly distributed in the central, southern, and eastern regions in China, indicating regional disparity of HPs in terms of population. By 2023 (Figure 1B), the clusters remained unchanged, with the exception of one high-value outlier that emerged within the low–low clustering region—Fujian (high–low type).

Figure 1C and Figure 1D present the distribution of HPs by geographical area in 2012 and 2023, respectively. High–high clustering was observed in East China (Shandong, Jiangsu), while low–low clustering was distributed in Northwest China (Xinjiang, Qinghai, etc.) and Southwest China (Tibet, Sichuan, etc.) in 2012. Additionally, three low-value outliers (classified as low–high type) were identified (Hebei). By 2023 (Figure 1D), the spatial patterns of high–high and low–low clusters remained largely unchanged, while the number of low-value outliers decreased to two (Anhui, Zhejiang).

A total of 24 figures were visualized for 12 years (2012–2023) (Figures S4 and S5). As shown in Table S3, the trend of HPs by population in Fujian Province changed from a low–low to a high–low pattern from 2012 to 2023. Twelve low–low type regions were identified in 2012, of which 10 regions still displayed a similar pattern in 2023. No low–high type of distribution of HPs by population was detected in the last decade. HPs by geographical area in Hebei changed from a low–high type in 2012 to a non-significant type in 2023. No high–low type of distribution of HPs by geographical area was detected in the last decade.

### 3.4. Analysis of Influencing Factors Based on Geographic Detector Model

The distribution of HPs per thousand population is relatively equitable; however, disparities persist when examining distribution across geographic regions. Consequently, the geographic detector is employed to analyze the factors influencing the spatial variation in HPs with respect to regional differences. Since 2021, no publicly available data on maternal mortality rates and average life expectancy across the 31 provinces have been released. The analysis in this section is therefore based on data from 2020.

As shown in Table 5, among the six factors passing the significance test at a 5% level, the percentage of population with a university degree or above (q = 0.9402) showed the largest explanatory power for the allocation of HPs in CDC sites by geographical area. Additional factors included residents’ healthcare expenditure (q = 0.7542), per capita GDP (q = 0.8052), life expectancy (q = 0.5822), per capita disposable income (q = 0.8062) and proportion of urban population (q = 0.7342).

**Figure 2:**
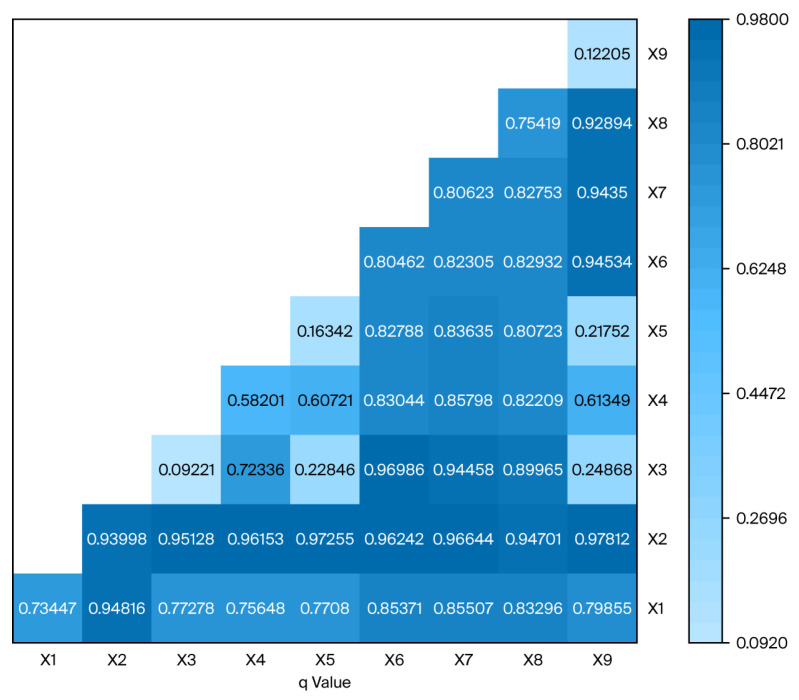
Results of the interactive detection (based on geographical area).

The results showing the interaction between factors are presented in Figure 2. The pairwise interactions among the multiple factors reveal that the interaction q-values for all factor pairs exceed those of their corresponding individual factors, indicating that synergistic effects between any two factors enhance the explanatory power regarding the spatial heterogeneity in the geographical distribution of health personnel. Among the drivers affecting HP distribution per square kilometer, the percentage of population with a university degree or above (x2) had a maximum q value via interaction with other factors. Although the proportion of older population (x3) alone was not a significant factor, its interaction with per capita GDP (x6) and per capita disposable income (x7) increased the explanatory power, which rose by approximately 0.95. This suggests that its influence is primarily mediated through synergistic interactions with other socioeconomic factors.

### 3.5. Robustness Testing

Comparative analyses were performed in this study using different model parameters and classification methods to evaluate the sensitivity of the discretization process for continuous independent variables in the geodetector model. This study implemented two complementary robustness assessments: (1) K-means clustering was applied with varying numbers of clusters (K = 3, 4, 5); (2) the interquartile range (IQR) method were also implemented as an alternative discretization scheme. Table S4-1 summarizes the factor detection outcomes obtained from K-means clustering across a range of K values. Table S4-2 summarizes the results of factor detection analysis following discretization of the variables via the quartile-based method. The results consistently identified the same key influencing factors, with minimal variation in their relative explanatory strength, confirming that the model outcomes are insensitive to parameter selection and discretization strategies. Collectively, the robustness tests support the reliability and consistency of the study’s main conclusions.

## 4. Discussion

Firstly, the Gini coefficient based on the population dimension and geographical area indicated different allocation of HPs among CDC sites in China. The distribution based on population was relatively equitable, whereas the geographical distribution showed significant disparity across different regions. This finding is consistent with the results of other studies on the health workforce; for example, Lingying Wang et al. [24] applied the Gini coefficient and Theil index to evaluate the equity of healthcare technician and nurse allocation between Chinese hospitals and primary healthcare facilities, and concluded that population-based allocation was equitable while geographical area-based allocation showed relative inequity. This difference may be related to the long-term allocation strategy of health resources based on the population in China [25]. Areas with better economic conditions tend to have larger population density; therefore, the number of HPs allocated by population is lower. In contrast, larger regions tend to be sparsely populated, with a lower number of HPs based on geographical area. Thus, provinces with relative equity by population allocation have low equity based on area allocation. Similar studies were almost unanimous in reporting that the distribution of HPs, including physicians, nurses and pharmacists, was strongly unequal based on geographical area compared to population [24,26,27]. For example, Ola Al Eker and Asma Imam [27] used the Gini coefficient to assess the equity of resource distribution between hospitals and primary healthcare centers in the West Bank region of Palestine. They found that equity based on population was significantly higher than that based on geographical area. These studies indicated that the uneven geographical distribution of health professionals may be a widespread phenomenon globally.

Based on the spatial analysis of the HP distribution among CDC sites in 2023, four typical regional patterns can be identified. The number of HPs per thousand people and per square kilometer is below the national average (Shanxi, Guangdong, Chongqing, etc.), representing a dual challenge that can be characterized as a “dual-deficit” type, which is the most prevalent category. The second category is characterized by insufficient geographical coverage (e.g., Xinjiang, Xizang and Inner Mongolia). The third category exhibits insufficient population coverage (e.g., Shanghai, Tianjin and Jiangsu). The final category, referred to as the “dual equilibrium type” is defined by values above the national average for both the number of HPs per thousand people and per square kilometer (e.g., Beijing and Fujian). This pattern clearly reflects the practical challenges associated with the distribution of HPs in achieving efficient population service delivery and equitable geographical distribution.

Secondly, the results of spatial correlation analysis showed an obvious spatial aggregation in the distribution of CDC HPs in China. From 2012 to 2023, the spatial aggregation of HPs by population distribution gradually weakened (global Moran’s I index from 0.503 to 0.238). This development partially reflects the positive outcomes of population-centered health policies. In contrast, spatial clustering based on geographical distribution exhibited a slight increase, albeit remaining low overall (global Moran’s I ranging from 0.137 to 0.151), suggesting that HPs have remained broadly dispersed across regions without forming prominent high-density clusters. The marginal rise in geographic clustering further implies a potential entrenchment of regional imbalances. This divergence underscores a critical challenge: despite improvements in population-proportional equity, remote and sparsely populated areas continue to face significant gaps in the geographical accessibility of health workforce resources.

The local Moran’s I index further elucidates the spatial pattern characteristics of the distribution of HPs across China. Geographically, high–high clustering is consistently observed in eastern provinces such as Shandong and Jiangsu, whereas low–low clustering persists in northwestern and southwestern regions, including Xinjiang, Tibet and Qinghai. This spatial distribution is closely associated with regional economic development levels. This finding regarding the distribution of the health workforce and economic level is consistent with the results reported by McDonald et al. in an Australian study [28]. It is important to emphasize that the observed spatial clustering of HPs and its association with regional economic development levels, as identified in this study, do not imply a unidirectional causal effect of economic factors on health workforce distribution. A potential reverse causal relationship cannot be ruled out. Simultaneously, the use of provincially aggregated data may introduce omitted variable bias, particularly with respect to relevant policy covariates, and is subject to inherent limitations associated with ecological inference. Consequently, the observed spatial concordance between economic indicators and human resource distribution reflects a statistical association only. The directionality, magnitude, and underlying mechanisms of any potential causal relationship require rigorous investigation through longitudinal, multilevel, or quasi-experimental designs. On one hand, the eastern region benefits from robust economic capacity, advanced urbanization, and substantial public health investment, resulting in a high density of the health workforce per unit area. On the other hand, the western region is characterized by vast territorial expanse, complex topography, and dispersed population settlements, which pose significant challenges to the spatial coverage of health resources. The persistent “low–low” spatial clustering observed in western China may also reflect the adverse impact of health workforce outmigration, driven primarily by disparities in material and socioeconomic conditions. Kuhlmann et al. [29] identified salary disparities and limited career advancement opportunities as the primary determinants of health workforce migration in their systematic analysis of global health labor mobility. Low-income countries frequently experience a paradoxical surplus: although they train substantial numbers of health professionals, retention remains critically low due to large-scale emigration to high-income countries. In China, the western regions face a relative shortage of health technicians, which can be attributed in part to substantial disparities in salary levels, career advancement prospects, and welfare benefits compared with the eastern regions. Despite potentially favorable per capita ratios of HPs, geographical accessibility consequently remains limited. It is critical to emphasize that while the local Moran’s I statistic identifies spatial heterogeneity, it does not serve as a direct indicator of service adequacy or quality.

Thirdly, based on the results of GDM in this study, factors such as population structure, population health level, economic development, and healthcare expenditures had a partial effect on geographical distribution of HPs in CDC sites. The urbanization rate, the proportion of the population with a college education or above, life expectancy, per capita GDP, per capita disposable income and residents’ healthcare expenditure were confirmed to have a substantial influence on the distribution of HPs. The findings are consistent with serval studies. Qian Bai et al., applying a spatial Durbin panel model, confirmed that the urbanization rate and government health expenditure exert a significant positive impact on local nurse allocation [30]. Similarly, Yingying Yu et al. found that demographic structure (e.g., urbanization rate and proportion of elderly population) and health indicators (e.g., incidence of infectious diseases) significantly affect the distribution of CDC resources [31]. Employing dynamic convergence and fixed-effects models, Afei Qin et al. demonstrated that per capita GDP growth has a significant nonlinear effect on the regional convergence speed of physician distribution [32]. Similar to the spatial clustering analysis, the correlation identified herein reflects an associative relationship. The geographical detector method detects spatial associations rather than establishing causal relationships.

Finally, the pairwise interaction generated a stronger explanatory power than the single factor for HP allocation. Among the various pairwise interactions, the proportion of the population with a college education or above and the proportion of older population—two key regional demographic characteristics—exhibit pronounced interaction effects when combined with other factors, particularly economic indicators, thereby substantially enhancing the explanatory power of individual variables. The most significant interaction was observed between the proportion of the population with a college education or above and health expenditure as a percentage of GDP (q = 0.9781), suggesting that the synergy between higher educational attainment and robust health investment can be more effectively translated into improved efficiency in health human resource allocation. Furthermore, the interaction between the older population ratio and per capita GDP demonstrates a markedly increased explanatory capacity (q = 0.9699), indicating that in regions with advanced population aging, a higher level of economic development can mitigate the adverse impacts of demographic aging on the distribution of HPs, with economic strength serving as a critical enabler in addressing structural population challenges. Although this study demonstrates that macroeconomic indicators—such as per capita GDP and per capita disposable income—exert a positive influence on the geographic allocation of health professionals, these aggregate measures fail to capture micro-level dynamics, including individual retention intentions and attrition risks. Notably, even in economically disadvantaged provinces where the population-adjusted distribution ratio of health professionals appears adequate, persistent out-migration may result in acute shortages of actively deployed personnel. Future research should consequently integrate granular, primary survey data to rigorously quantify how compensation levels, benefit packages, and career advancement opportunities shape the retention intentions of public health professionals—particularly those working in disease prevention and control institutions.

These findings collectively underscore that the geographical distribution of HPs is shaped by the interplay of multiple determinants, including demographic composition, educational advancement, economic conditions, and health policy investments. Therefore, future strategies for optimizing human resource allocation in public health should emphasize the synergistic effects of integrated policy interventions. Specifically, comprehensive approaches involving the enhancement of educational standards, strengthening of economic foundations and strategic optimization of health investment structures are essential to systematically promoting equitable spatial distribution of the health workforce. Based on the findings and implications of this study, we propose the following evidence-informed policy recommendations. First, within the framework of the 15th Five-Year Plan, priority should be accorded to designing and implementing targeted recruitment and retention incentive mechanisms specifically for public health professionals in underserved regions. Second, the statistically significant interaction between educational attainment and health expenditure indicates that unilateral increases in health investment yield diminishing returns; rather, such investment must be strategically coupled with enhancements in local medical education capacity and sustained talent development initiatives. Third, the robust interaction between population aging ratio and per capita GDP underscores that economic development serves as a critical mitigating factor against demographic pressures in rapidly aging areas. Consequently, human resource planning and allocation models should integrate both aging indicators and per capita GDP as co-determinants replacing rigid, uniform staffing standards with context-sensitive, dynamic calibration approaches. Fourth, the pronounced interdependence among education level, economic conditions, and health system performance necessitates institutionalized cross-sectoral coordination. Specifically, ministries responsible for education, economic development, and health must jointly formulate integrated human resource planning objectives, moving beyond siloed policymaking toward coherent, system-wide governance.

The study has some limitations. First, we only assessed the equity and spatial pattern of HPs, while other types of health human resources may present different characteristics that require further analysis. The Gini coefficient is also limited in its ability to capture nuances in the allocation of health resources. Second, only the resident population was used to assess the equity of population distribution, which may not reflect the real-life data due to migration or local policy interventions. Thirdly, due to the limited data available, we only discussed the distribution of HPs in CDC sites at the provincial level. The distribution of HPs at the municipal and district levels needs further analysis. Fourth, Descriptive analysis encompasses the full panel dataset spanning 2012–2023, whereas the geographical detector-based factor analysis is conducted exclusively on the 2020 cross-sectional data. Consequently, the identified explanatory factors capture spatial associations specific to 2020 and do not generalize across the entire study period. Finally, several indicators reported in the statistical yearbooks are derived from sample surveys rather than full censuses. Publicly available data also suffer from temporal lags and insufficient spatial granularity—limitations that may obscure sub-provincial or sub-institutional variations in the distribution of health workforce personnel. The yearbook data employed in this study capture only the headcount of on-duty personnel and do not differentiate between permanent and non-permanent staff, nor do they support the tracking of interprovincial or interinstitutional mobility pathways. Consequently, the present analysis may have some potential biases, such as those associated with the process of data collection, the diversity issues of data and the limited level of data available. Future research could advance understanding in this field based on the incorporation of additional factors covering a more precise area, using more advanced and reasonable methods, and the integration of complementary data sources.

## 5. Conclusions

China exhibited overall upward trends in CDC HPs per 1000 population and per square kilometer over the past 12 years. However, the study revealed a persistent inequity of CDC HP distribution by geographical area, with significant differences across regions from 2012 to 2023. In addition, the distribution of CDC HPs was more equitable based on population than on geographical location in China. The distribution of HPs in CDC institutions showed spatial aggregation, with the distribution by geographical area remaining persistently uneven and showing minimal improvement over the past 12 years. These findings, derived from descriptive inequality metrics and spatial correlation analysis, do not establish causal relationships. Moreover, incorporating geographical equity as a policy objective entails inherent normative constraints. We recommend that the distribution policy of CDC health professionals maybe take into account more factors, including the geographical area, population education levels, aging demographics, regional economic development and local health expenditures, rather than just population size. Adopting integrated measures will be crucial to systematically reducing spatial inequities in the allocation of HPs. However, the causal relationship remains to be empirically validated.

## Figures and Tables

**Table 1:** Definitions and measurements of variables.

Factor	Specific Indicator	Definition	Factor Code
Demographic structure	Proportion of urban population (%)	Proportion of urban population in the total population	x1
	Percentage of population with a university degree or above (%)	Proportion of population with college degree and above in the total population	x2
	Proportion of older population (%)	Proportion of the population aged 65 and over in the total population	x3
Population health level	Life expectancy (years)	Average age of survival from birth in a population group	x4
	Maternal mortality rate (1/100,000)	Number of maternal deaths during a given time period per 100,000 live births during the same time period	x5
Economic development	Per capita GDP (CNY)	GDP values (CNY 10,000) divided by the population size at the end of the year in each province	x6
	Per capita disposable income (CNY)	Individual income remaining after deduction of taxes and social insurance contributions paid to the government.	x7
Health expenditure	Residents’ healthcare expenditure (CNY)	Average annual expenditure per capita on medicines, medical devices, and healthcare services.	x8
	Health expenditure as a percentage of GDP (%)	Total health expenditure divided by GDP for the same year in each province	x9

**Note:** x1–x9 are selected from https://www.stats.gov.cn/sj/ndsj/ (accessed on 31 October 2025). The interpretive analysis in this study employed the Geodetector model and was conducted using 2020 cross-sectional provincial data.

**Table 2:** Distribution of CDC HPs in China from 2012 to 2023.

Year	Population (10,000 Persons)	Total Area (km^2^)	Number of HPs (Person)	Density of HPs	
				Per 1000 Persons	Per Square Kilometer
2012	135,694	9,670,711	141,261	0.1041	0.0146
2013	136,496	9,670,711	143,101	0.1048	0.0148
2014	137,417	9,670,711	142,297	0.1036	0.0147
2015	138,097	9,670,711	141,698	0.1026	0.0147
2016	139,003	9,670,711	142,492	0.1025	0.0147
2017	139,779	9,670,711	142,114	0.1017	0.0147
2018	140,310	9,670,711	140,491	0.1001	0.0145
2019	140,805	9,670,711	139,839	0.0993	0.0145
2020	141,013	9,670,711	145,229	0.1030	0.0150
2021	141,060	9,670,711	158,475	0.1123	0.0164
2022	140,975	9,670,711	168,909	0.1198	0.0175
2023	140,767	9,670,711	181,441	0.1289	0.0188
Growth rate (%)			28.44%	23.82%	28.77%

**Table 3:** The Gini coefficients of HP distribution at CDC sites from 2012 to 2023.

Year	Distribution Based on Population	Distribution Based on Geographical Area
2012	0.1577	0.5809
2013	0.1598	0.5862
2014	0.1564	0.5803
2015	0.1576	0.5794
2016	0.1579	0.5796
2017	0.1629	0.5760
2018	0.1635	0.5744
2019	0.1643	0.5741
2020	0.1605	0.5773
2021	0.1642	0.5707
2022	0.1548	0.5782
2023	0.1624	0.5848
Average Gini coefficient	0.1602	0.5785

**Table 4:** The global Moran’s I of CDC health professional distribution.

Year	Distribution Based on Population	*p*-Value	Z-Value	Distribution Based on Geographical Area	*p*-Value	Z-Value
2012	0.503	<0.01	5.020	0.137	0.016	2.144
2013	0.468	<0.01	4.851	0.139	0.015	2.161
2014	0.451	<0.01	4.742	0.128	0.017	2.112
2015	0.443	<0.01	4.686	0.135	0.017	2.119
2016	0.462	<0.01	4.803	0.130	0.018	2.097
2017	0.450	<0.01	4.862	0.129	0.018	2.087
2018	0.446	<0.01	4.867	0.130	0.019	2.085
2019	0.415	<0.01	4.609	0.137	0.016	2.147
2020	0.406	<0.01	4.463	0.162	0.009	2.384
2021	0.404	<0.01	4.541	0.159	0.007	2.479
2022	0.348	<0.01	3.937	0.161	0.007	2.461
2023	0.238	<0.01	2.886	0.151	0.008	2.389

**Table 5:** Results of the factor detection.

		x1	x2	x3	x4	x5	x6	x7	x8	x9
Allocation based on geographical area	q	0.734	0.940	0.092	0.582	0.163	0.805	0.806	0.754	0.122
	*p*	<0.001	<0.001	0.474	0.003	0.205	<0.001	<0.001	<0.001	0.390

Note: x1: Proportion of urban population (%); x2: Percentage of population with a university degree or above (%); x3: Proportion of older population (%); x4: Life expectancy (years); x5: Maternal mortality rate (1/100,000); x6: Per capita GDP (CNY); x7: Per capita disposable income (CNY); x8: Residents’ healthcare expenditure (CNY); x9: Health expenditure as a percentage of GDP (%).

## Data Availability

The datasets generated during the current study are available from following websites: http://www.nhc.gov.cn/mohwsbwstjxxzx/tjzxtjsj/tjsj_list.shtml (accessed on 31 October 2025); https://www.stats.gov.cn/sj/ndsj/ (accessed on 31 October 2025); and https://www.ngcc.cn/ (accessed on 31 October 2025). The survey data collected and analyzed during the current study are also available from the corresponding author on reasonable request.

## Supplementary Materials

The following supporting information can be downloaded at: https://www.mdpi.com/article/10.3390/healthcare14081079/s1, Figure S1: Personnel density from 2012 to 2023 in China; Figure S2: Gini coefficient from 2012 to 2023 in China; Figure S3: Global Moran’s I from 2012 to 2023 in China; Figure S4: LISA map based on geographical area; Figure S5: LISA map based on population; Table S1: Provincial distribution of CDC health professional density in China in 2023; Table S2: Driving factors in 2020; Table S3: Classification of four types of spatial autocorrelation for CDC HPs in 2012 and 2023; Table S4-1: Results of the factor detection (k-means); Table S4-2: Results of the factor detection (quartile method).
