# Supplementary material for: Regional Disparities and Associated Factors Underlying CDC Health Professional Distribution in China
Source: Healthcare (Basel). 2026 Apr 17;14(8):1079. doi: 10.3390/healthcare14081079 (PMC13115888; doi:10.3390/healthcare14081079)
Supplement: Supplementary file 1 [file healthcare-14-01079-s001.zip › healthcare-4174819-supplementary.pdf]

Table S1. Provincial distribution of CDC health professional density in China in 2023

Province	Population (10,000 persons)	Total area (km^2)	Health Professional (person)	Density of HPs	
				Per 1,000 persons	Per square kilometer
Beijing（BJ）	2186	16,410	3631	0.1661	0.2213
Tianjin（TJ）	1364	11,967	1929	0.1414	0.1612
Hebei（HeB）	7393	188,800	7830	0.1059	0.0415
Shanxi（SX）	3466	156,700	3515	0.1014	0.0224
Inner Mongolia（IM）	2396	1,183,000	6373	0.2660	0.0054
Liaoning（LN）	4182	148,000	5634	0.1347	0.0381
Jilin（JL）	2339	187,400	3389	0.1449	0.0181
Heilongjiang（HLJ）	3062	473,000	5285	0.1726	0.0112
Shanghai（SH）	2487	6,340	3017	0.1213	0.4759
Jiangsu（JS）	8526	107,200	9308	0.1092	0.0868
Zhejiang（ZJ）	6627	105,500	5775	0.0871	0.0547
Anhui（AH）	6121	140,100	7412	0.1211	0.0529
Fujian（FJ）	4183	124,000	9069	0.2168	0.0731
Jiangxi（JX）	4515	166,900	5367	0.1189	0.0322
Shandong（SD）	10123	158,000	11551	0.1141	0.0731
Henan（HeN）	9815	167,000	11087	0.1130	0.0664
Hubei（HuB）	5838	185,900	8101	0.1388	0.0436
Hunan（HuN）	6568	211,800	7680	0.1169	0.0363

Continued Table S1. Provincial distribution of CDC health professional density in China in 2023

Province	Population (10,000 persons)	Total area (km^2)	Health Professional (person)	Density of HPs	
				Per 1,000 persons	Per square kilometer
Guangdong（GD）	12706	179,725	9064	0.0713	0.0504
Guangxi（GX）	5027	237,600	6912	0.1375	0.0291
Hainan（HaN）	1043	35,400	1354	0.1298	0.0382
Chongqing（CQ）	3191	82,402	2984	0.0935	0.0362
Sichuan（SC）	8368	486,000	11636	0.1391	0.0239
Guizhou（GZ）	3865	176,167	5697	0.1474	0.0323
Yunnan（YN）	4673	394,100	8422	0.1802	0.0214
Tibet（TB）	365	1,228,400	1508	0.4132	0.0012
Shaanxi（SaX）	3952	205,600	5242	0.1326	0.0255
Gansu（GS）	2465	453,700	4187	0.1699	0.0092
Qinghai（QH）	594	722,300	1230	0.2071	0.0017
Ningxia（NX）	729	66,400	1029	0.1412	0.0155
Xinjiang（XJ）	2598	1,664,900	6223	0.2395	0.0037

Table S2. Driving factors in 2020

Province	x1	x2	x3	x4	x5	x6	x7	x8	x9
Beijing（BJ）	87.55	28.61	13.30	82.49	4.80	164889	69433.5	3513.3	8.39
Tianjin（TJ）	84.7	15.96	14.75	81.30	7.50	101614	43854.1	2646.0	6.44
Hebei（HeB）	60.07	5.35	13.92	77.75	9.30	48564	27135.9	1692.0	8.48
Shanxi（SX）	62.53	8.02	12.91	77.91	11.00	50528	25213.7	1854.0	8.38
Inner Mongolia（IM）	67.48	8.72	13.06	77.56	14.90	72062	31497.3	1891.5	7.30
Liaoning（LN）	72.14	9.37	17.43	78.68	10.90	58872	32738.3	2303.2	7.60
Jilin（JL）	62.64	9.24	15.66	78.41	11.50	50800	25751.0	2031.2	9.54
Heilongjiang（HLJ）	65.61	7.50	15.68	78.25	15.40	42635	24902.0	2023.2	12.97
Shanghai（SH）	89.3	21.51	16.27	82.55	7.40	155768	72232.4	3033.4	6.81
Jiangsu（JS）	73.44	9.00	16.19	79.32	5.10	121231	43390.4	2018.6	4.79
Zhejiang（ZJ）	72.17	8.50	13.24	80.19	3.90	100620	52397.4	1955.9	5.91
Anhui（AH）	58.33	6.05	15.00	77.96	7.10	63426	28103.2	1548.0	6.30
Fujian（FJ）	68.75	7.15	11.08	78.49	9.20	105818	37202.4	1583.2	4.39
Jiangxi（JX）	60.44	5.17	11.89	77.64	5.90	56871	28016.5	1437.3	6.99
Shandong（SD）	63.05	6.65	15.11	79.18	6.80	72151	32885.7	1914.0	6.60
Henan（HeN）	55.43	4.90	13.48	77.60	9.50	55435	24810.1	1621.9	7.15
Hubei（HuB）	62.89	7.41	14.66	78.00	10.00	74440	27880.6	1764.9	7.94
Hunan（HuN）	58.76	5.14	14.81	77.88	9.30	62900	29379.9	2034.7	6.89
Guangdong（GD）	74.15	7.10	8.57	79.31	6.10	88210	41028.6	1677.9	6.39
Guangxi（GX）	54.2	4.74	12.18	78.06	8.40	44309	24562.3	1540.7	8.47

Continued Table S2. Driving factors in 2020

Province	x1	x2	x3	x4	x5	x6	x7	x8	x9
Hainan（HaN）	60.27	6.59	10.39	79.05	15.10	55131	27904.1	1407.3	9.58
Chongqing（CQ）	69.46	7.25	17.06	78.56	9.50	78170	30823.9	2101.5	6.24
Sichuan（SC）	56.73	6.04	16.92	77.79	9.40	58126	26522.1	1908.0	8.32
Guizhou（GZ）	53.15	5.37	11.55	75.20	15.90	46267	21795.4	1269.6	8.36
Yunnan（YN）	50.05	5.51	10.74	74.02	12.40	51975	23294.9	1547.4	7.79
Tibet（TB）	35.73	5.82	5.65	72.19	47.90	52345	21744.1	589.9	11.06
Shaanxi（SaX）	62.66	8.77	13.32	77.80	7.70	66292	26226.0	2078.4	7.75
Gansu（GS）	52.23	6.79	12.59	75.64	15.10	35995	20335.1	1544.7	11.26
Qinghai（QH）	60.08	7.07	8.67	73.96	24.90	50819	24037.4	1975.7	12.71
Ningxia（NX）	64.96	8.37	9.61	76.58	11.20	64528	25734.9	1906.3	9.64
Xinjiang（XJ）	56.53	6.73	7.74	75.65	17.00	53593	23844.7	1611.7	10.95

Note:x1: Proportion of urban population (%); x2: Percentage of population with a university degree or above (%);x3:Proportion of the aged population(%);x4: Life expectancy (year); x5: Maternal mortality rate (‰$_{000}$); x6: per capital GDP (CNY); x7: Per capita disposable income (CNY); x8: Residents' health care expenditure (CNY); x9: Health expenditure as a percentage of GDP (%).

Table S3. Classification of four types of spatial autocorrelation for CDC HPs in 2012 and 2023

Year	Type	Number	Allocation by population	Number	Allocation by geographical
2012	High–high	2	XJ, TB	2	JS, SD
	High–low	0	none	0	none
	Low–high	0	none	3	HeB, AH, ZJ
	Low–low	12	JS, ZJ, AH, FJ, JX, HeN, HuB, HuN, GD, GX, CQ, GZ	5	XJ, TB, GS, QH, SC
2023	High–high	2	XJ, TB	2	JS, SD
	High–low	1	FJ	0	none
	Low–high	0	none	2	AH, ZJ
	Low–low	10	JS, AH, JX, SD, HeN, HuB, HuN, CQ, GZ, SaX	5	XJ, TB, GS, QH, SC

Table S4-1. Results of the factor detection(k-means)

	K		x1	x2	x3	x4	x5	x6	x7	x8	x9
Allocation based on geographical area	K=3	q	0.733	0.751	0.093	0.579	0.095	0.786	0.802	0.731	0.131
		p	< 0.001	< 0.001	0.291	< 0.001	0.260	< 0.001	< 0.001	< 0.001	0.175
	K=4	q	0.734	0.940	0.092	0.582	0.163	0.805	0.806	0.754	0.122
		p	< 0.001	< 0.001	0.474	0.003	0.205	< 0.001	< 0.001	< 0.001	0.390
	K=5	q	0.740	0.940	0.115	0.754	0.286	0.805	0.813	0.756	0.139
		p	< 0.001	< 0.001	0.559	< 0.001	0.102	< 0.001	< 0.001	< 0.001	0.473

Table S4-2. Results of the factor detection(quartile method)

		x1	x2	x3	x4	x5	x6	x7	x8	x9
Allocation based on geographical area	q	0.372	0.307	0.090	0.428	0.274	0.394	0.396	0.373	0.101
	p	0.026*	0.048*	0.513	0.011*	0.074	0.019*	0.018*	0.046*	0.455

*: $p < 0.05$

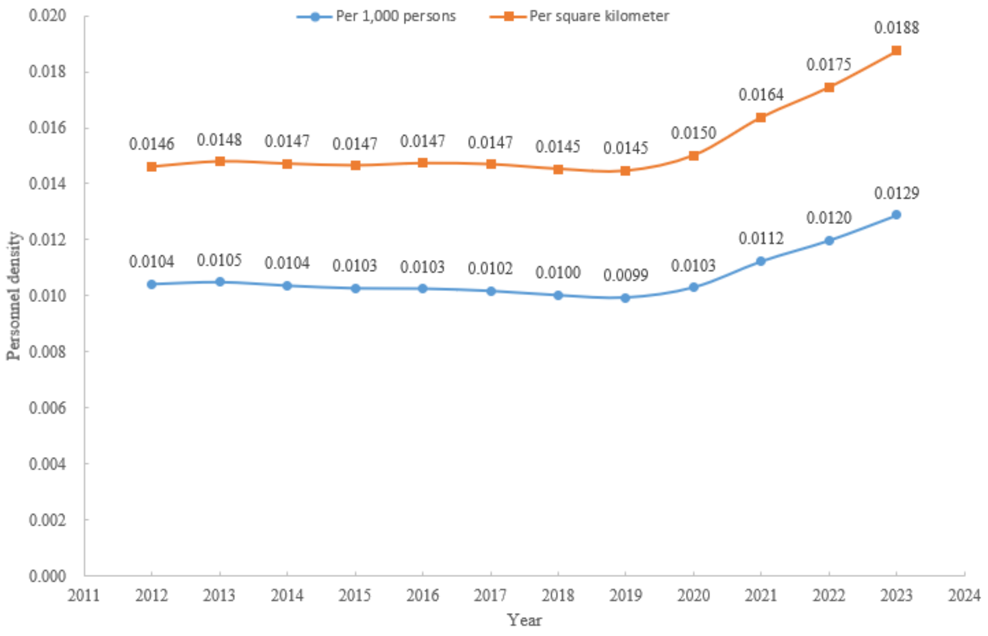

Figure S1. Personnel density from 2012 to 2023 in China

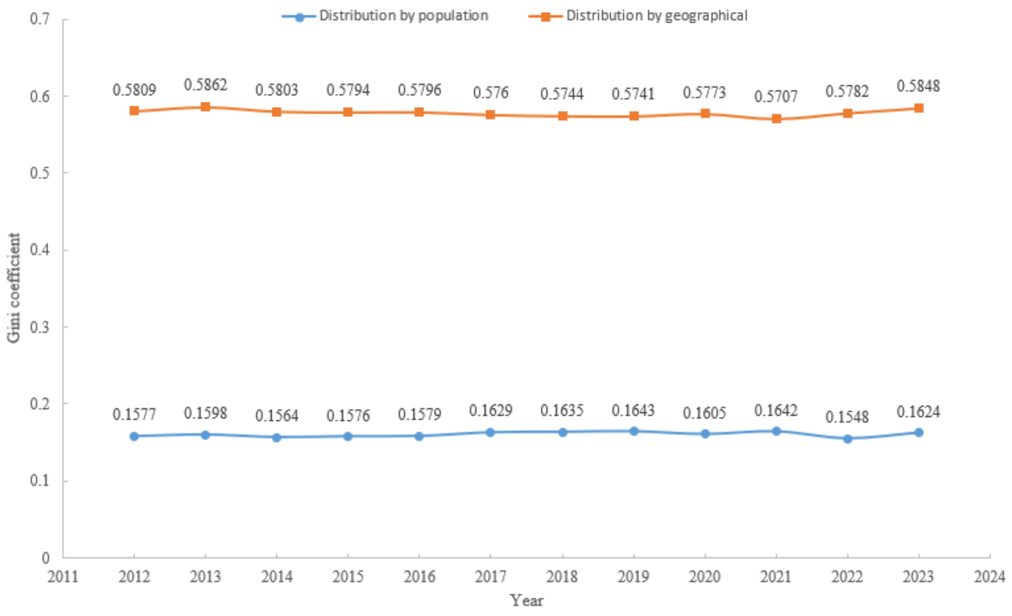

Figure S2. Gini coefficient from 2012 to 2023 in China

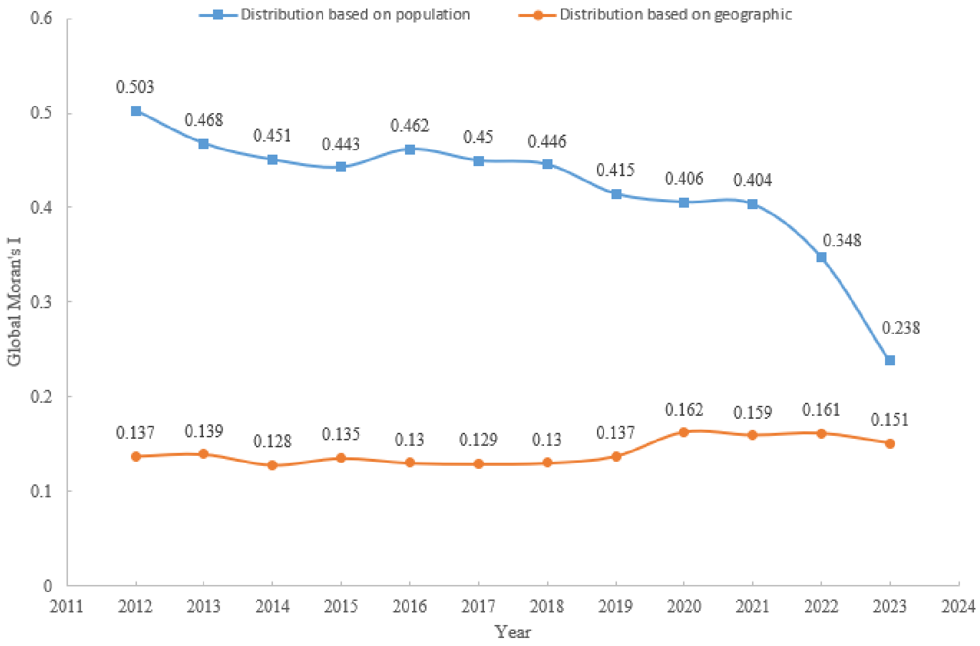

Figure S3. Global Moran's I from 2012 to 2023 in China

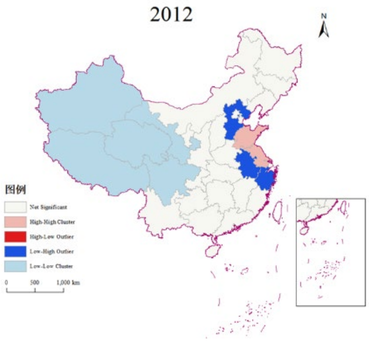

Figure S4-1 Lisa map in 2012

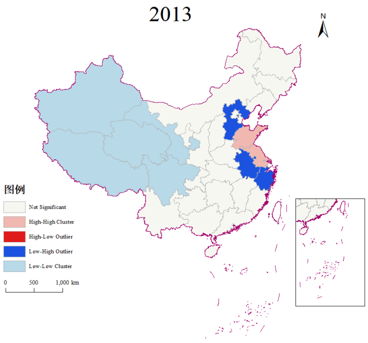

Figure S4-2 Lisa map in 2013

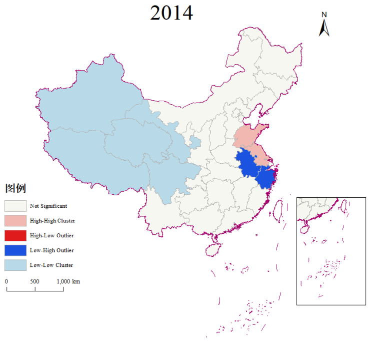

Figure S4-3 Lisa map in 2014

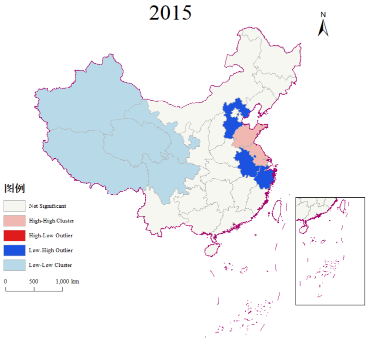

Figure S4-4 Lisa map in 2015

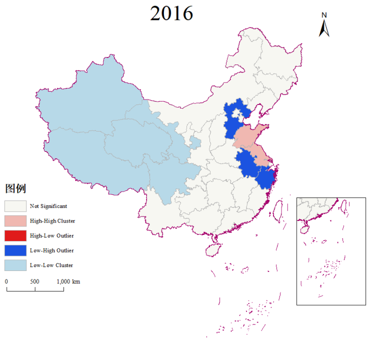

Figure S4-5 Lisa map in 2016

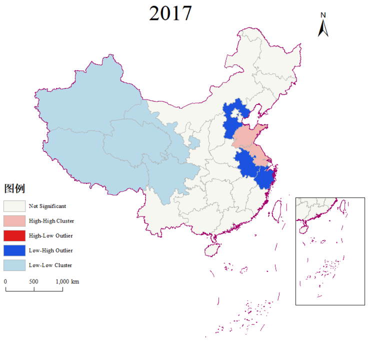

Figure S4-6 Lisa map in 2017

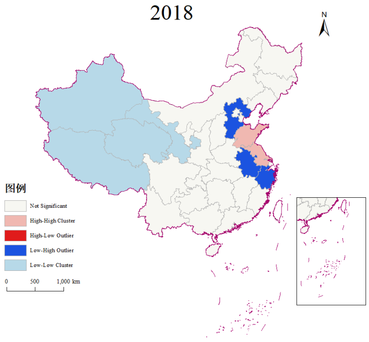

Figure S4-7 Lisa map in 2018

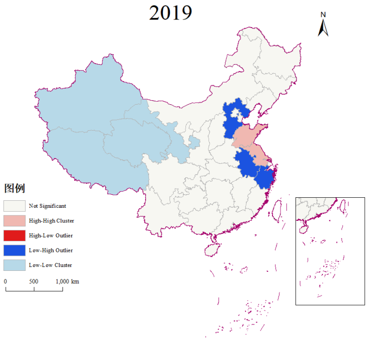

Figure S4-8 Lisa map in 2019

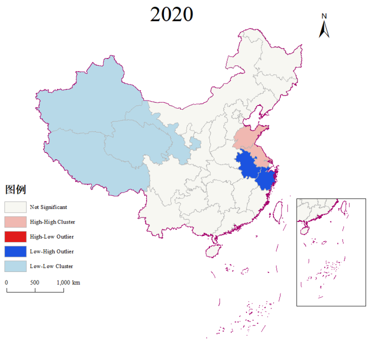

Figure S4-9 Lisa map in 2020

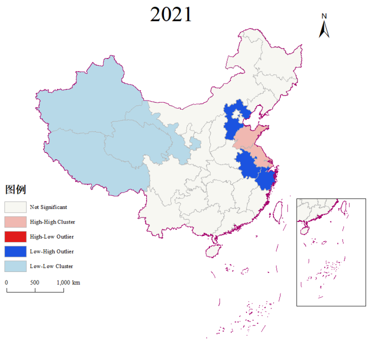

Figure S4-10 Lisa map in 2021

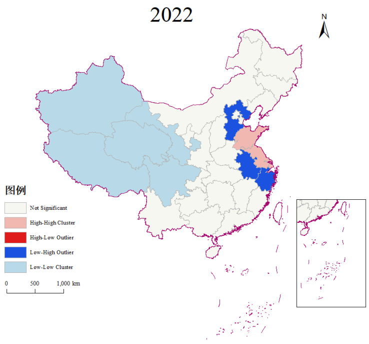

Figure S4-11 Lisa map in 2022

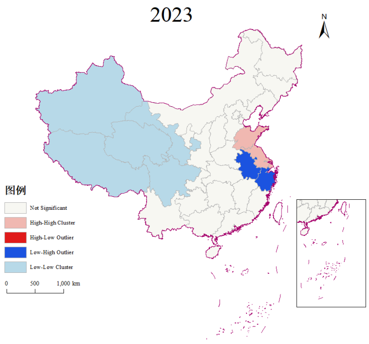

Figure S4-12 Lisa map in 2023

Figure S4. Lisa map-based on geographical area

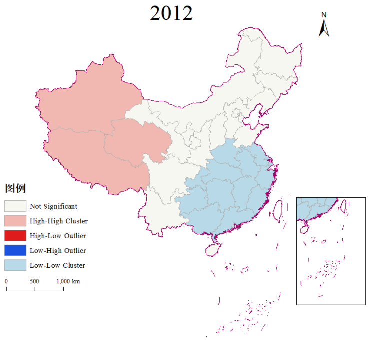

Figure S5-1 Lisa map in 2012

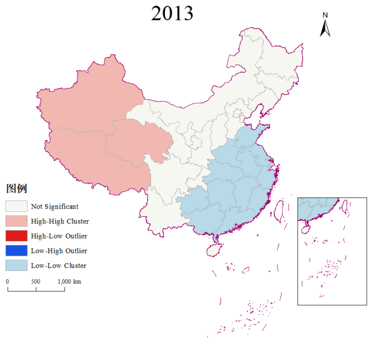

Figure S5-2 Lisa map in 2013

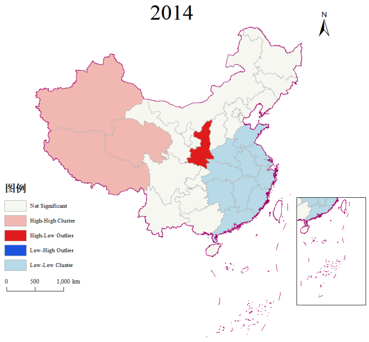

Figure S5-3 Lisa map in 2014

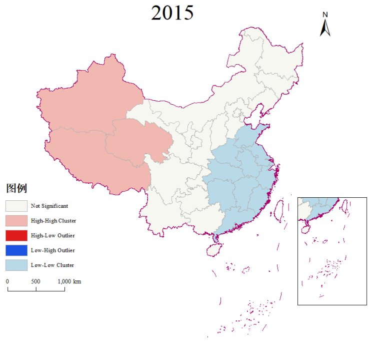

Figure S5-4 Lisa map in 2015

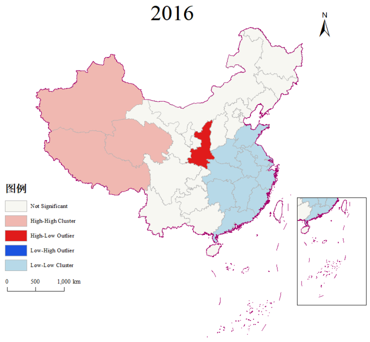

Figure S5-5 Lisa map in 2016

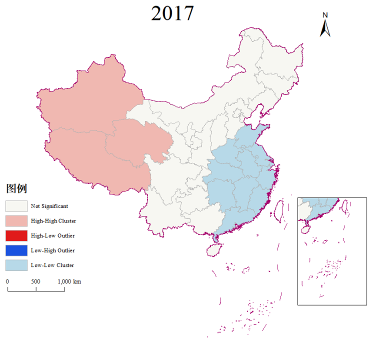

Figure S5-6 Lisa map in 2017

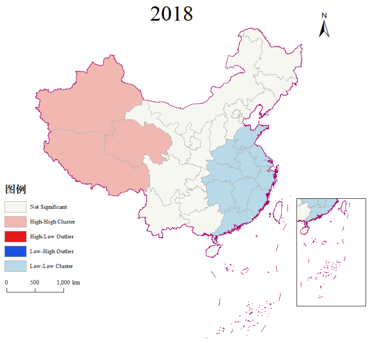

Figure S5-7 Lisa map in 2018

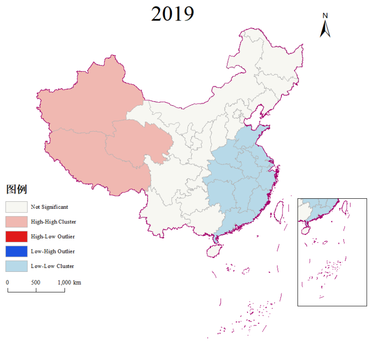

Figure S5-8 Lisa map in 2019

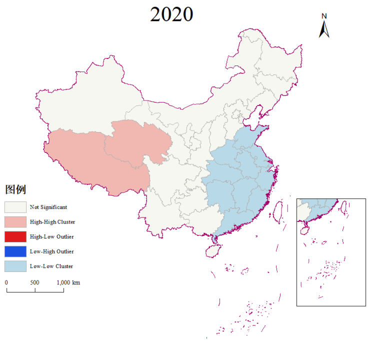

Figure S5-9 Lisa map in 2020

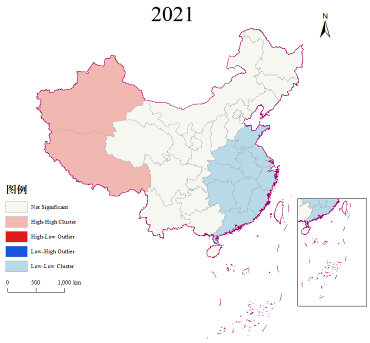

Figure S5-10 Lisa map in 2021

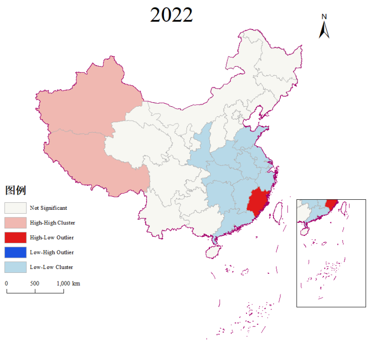

Figure S5-11 Lisa map in 2022

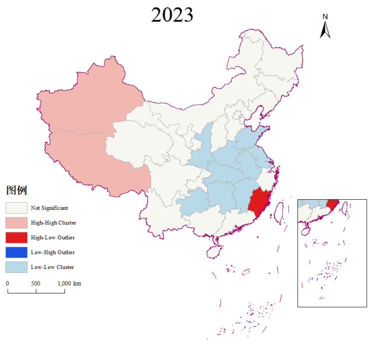

Figure S5-12 Lisa map in 2023

Figure S5. Lisa map-based on population
